# Enhancement of Radiation Effects by Ursolic Acid in BGC-823 Human Adenocarcinoma Gastric Cancer Cell Line

**DOI:** 10.1371/journal.pone.0133169

**Published:** 2015-07-15

**Authors:** Yang Yang, Man Jiang, Jing Hu, Xin Lv, Lixia Yu, Xiaoping Qian, Baorui Liu

**Affiliations:** The Comprehensive Cancer Centre of Drum Tower Hospital, Medical School of Nanjing University & Clinical Cancer Institute of Nanjing University, Nanjing, Jiangsu, China; Lawrence Berkeley National Laboratory, University of California, Berkeley, UNITED STATES

## Abstract

Recent research has suggested that certain plant-derived polyphenols, i.e., ursolic acid (UA), which are reported to have antitumor activities, might be used to sensitize tumor cells to radiation therapy by inhibiting pathways leading to radiation therapy resistance. This experiment was designed to investigate the effects and possible mechanism of radiosensitization by UA in BGC-823 cell line from human adenocarcinoma gastric cancer *in vitro*. UA caused cytotoxicity in a dose-dependent manner, and we used a sub-cytotoxicity concentration of UA to test radioenhancement efficacy with UA in gastric cancer. Radiosensitivity was determined by clonogenic survival assay. Surviving fraction of the combined group with irradiation and sub-cytotoxicity UA significantly decreased compared with the irradiation group. The improved radiosensitization efficacy was associated with enhanced G2/M arrest, increased reactive oxygen species (ROS), down-regulated Ki-67 level and improved apoptosis. In conclusion, as UA demonstrated potent antiproliferation effect and synergistic effect, it could be used as a potential drug sensitizer for the application of radiotherapy.

## Introduction

Despite a decline in incidence, gastric cancer is still considered one of the leading causes of cancer-related deaths worldwide. Surgery continues to be the mainstay of any curative treatment; however, approximately two-thirds of patients diagnosed with gastric cancer have unresectable locally advanced and/or metastatic disease. The 5-year survival rate of patients who were diagnosed with advanced loco-regional gastric cancer and have undergone curative resection remain low due to the high risk of local recurrence or distant metastases even if resection was believed to be curative.[1] A perspective randomized Intergroup (INT)-0116 trial (556 patients) and retrospective Korean experience (990 patients) showed a reduction in local recurrence rate with postoperative regional radiation coupled with chemotherapy and a higher survival rate in the adjuvant arm.[2] Radiotherapy is the main loco-regional control modality for unresectable gastric cancer.[3] The National Comprehensive Cancer Network (NCCN) guidelines recommend radiotherapy as a standard treatment for gastric cancer patients with a high risk of recurrence; [4] However, there are two major limits associated with treating gastric cancer with radiation: (1) intrinsic or acquired resistance to radiotherapy; (2) nonspecific toxicity towards gastric mucosa and surrounding normal tissues.[2,5]

To address these limitations, scholarly attempts have been made to search for an effective radiosensitizer from herbs with lower toxicity and higher efficacy. Recent research has suggested that many plant-derived polyphenols, including curcumin, flavopiridol, emodin, ursolic acid and etc, might be used to sensitize tumor cells to chemotherapeutic agents and radiation therapy by inhibiting the pathways leading to treatment resistance.[6,7,8] These agents have also been found to be protective from therapy-associated toxicities.[5] Ursolic acid (UA), a pentacyclic triterpene acid, which is considered one of the most promising chemopreventive agent for cancer, has been shown to prompt antitumor activities including inhibiting of tumor initiation and promotion.[9,10] It also induced tumor cell differentiation by regulation of the expression of differentiation-specific genes in mouse F9 teratocarcinoma cells.[11] In addition, there has been evidence demonstrating that UA produced an anti-angiogenic effect in chick chorioalantoic membrane and an anti-invasive activity in HT1080 human fibrosarcoma cells.[12,13]

However, prior research has yet to demonstrate whether UA can enhance the radiation effects in malignant neoplasm. The experiment was designed to explore the effects and possible mechanism in vitro of radiosensitization by UA in BGC-823 cell line from human adenocarcinoma gastric cancer to provide the theoretical support for the clinical use.

## Materials and methods

### Cell culture

BGC-823 cells were obtained from Shanghai Institute of Cell Biology (Shanghai, China) and stored in RPMI 1640 medium with 10% calf bovine serum at 37°C in a water-saturated atmosphere with 5% CO_2_.

### Cell cytotoxicity assay

Cytotoxicity was determined by MTT assay performed a published previously.[14] To illustrate, cells were plated at 8×10^3^ cells per well into 96-well plates and allowed to attach overnight. Then, the cells were treated with various concentrations of UA (0, 5, 6.25, 10, 12.5, 20, 40, 50ug/ml) for 48h in a minimum of three replicate wells. Each well was then added 20ul MTT, prepared by mixing 5mg MTT with 1ml normal saline, and incubated for an additional 4h. Medium in each well was replaced with 150ul DMSO. Absorbance was determined with a microplate reader (BIP-RAD) at 490nm. The blank control wells were used for zeroing absorbance. The inhibition rate (IR%) was calculated using the background-corrected absorbance by the following equation:

$$\text{IR}\%=\left({\text{A}}_{\text{untreated control well}}\;\text{-}\;{\text{A}}_{\text{experimental well}}\right)\;/\;{\text{A}}_{\text{untreated control well}})\;\times 100\%.$$

The IC_50_ was defined as the concentration required for 50% inhibition of cell growth.

### Cell grouping and in vitro ionizing radiation

Cells were randomly divided into 4 groups: control group (A), UA group (B), radiation therapy (RT) group (C), and combined group (D). The BGC-823 cells were cultured in 6-well plates, and then Group B and D were treated with UA, while Group C and D were irradiated using a 6-MeV electron beam linear accelerator (Elekta, Sweden).

### Clonogenic survival assay

Radiosensitivity was determined by clonogenic survival assay. Cells (500–2000 per well) were plated in 6-well plates and allowed to attach overnight. Then cells were treated with UA (0, 6.25 and 10ug/ml) for 24h and then exposed to increasing doses of RT (0, 2, 4, 6 and 8Gy). After 10–14 days of incubation, colonies consisted of more than 50 cells were observed. The colonies were washed carefully, stained with ethanol/crystal violet dye, and then counted. The plating efficiency (PE) was calculated as (mean colony counts / cells seeded), and surviving fraction (SF) was calculated as [mean colony counts / (cells seeded ×PE)], where PE was defined as (mean colony counts for un-irradiated controls / cells seeded). D_0_ values were calculated by a multi-target single-hit model [S = 1 - (1-e^-D/D0^) ^N^], which represented the average dose of a lethal exposure. Sensitizer enhancement ratio (SER) was calculated as D_0_ ratio between combination treatment and RT alone.

### Cell cycle analysis

The influence of cell cycle was analyzed using Propidium Iodide / RNase buffer (BD Pharmingen, San Jose, CA, USA) staining according to the manufacturer’s instructions. 2.5×10^5^ cells were plated in 6-well plates and allowed to attach overnight. Then, cells were treated with UA (0 and 10ug/ml) for 24h and then exposed to RT (0 and 2Gy) for 48h. Cells were collected by trypsinization, fixed in 70% ethanol at -20°C, washed in PBS, resuspended in 1ml of PBS containing 1mg/ml RNase and 50 ug/ml propidium iodide, incubated in the dark for 30min at 37°C, and analyzed by flow cytometry (FACScan, Becton Dickinson, Sunnyvale, CA, USA).

### Measurement of apoptosis

Cells quantification of apoptosis cells was evaluated using an Annexin-V-FITC Apoptosis Detection Kit (BD Pharmingen, San Jose, CA, USA) according to the manufacturer’s instructions. 2.5×10^5^ cells were plated in 6-well plates and allowed to attach overnight. Then, cells were treated with UA (0 and 10ug/ml) for 24h and then exposed to RT (0 and 2Gy) for 48h. Cells were collected by trypsinization, and resuspended in 500ul of binding buffer, and 5ul of Annexin-V-fluorescein isothiocyanate (FITC), and then, 5ul of propidium iodide (PI) were added. Analyses were performed with a flow cytometry.

### Detection of reactive oxygen species (ROS) generation

The concentrations of intracellular ROS were detected using the membrane-permeable fluorescent probes 2’,7’-dichlorofluorescin diacetate (DCFH-DA) (Beyotime, Haimen, Jiangsu, China) according to the manufacturer’s instructions. 1×10^6^ cells were plated in 6-well plates and allowed to attach overnight. Then, cells were treated with UA (0 and 10ug/ml) for 24h, and then exposed to RT (0 and 2Gy) for 48h. Cells were collected by trypsinization, resuspended DCFH-DA for 30min, washed by serum-free RPMI 1640, and then detected by flow cytometry. The mean fluorescent intensity (MFI) represented intracellular ROS level.

### Immunohistochemical staining for assessing Ki-67 expression

Ki-67 protein expression was evaluated by the avidin-biotin complex immunohistochemical (ZSGB-BIO, Beijing, China) according to the manufacturer’s instructions. 2×10^4^ cells were plated in 6-well plates with a cover slip for each well, and allowed to attach overnight. Then cells were treated with UA (0 and 10ug/ml) for 24h, and then exposed to RT (0 and 2Gy) for 24h. Then the cover slips were fixed in 4% paraformaldehyde, incubated with 0.5% Triton X-100 for 20min, washed in PBS, and incubated with 3% H_2_O_2_ for 15min. After rinsing with PBS for three times, cells which were attached on the cover slips were incubated with Ki-67 monoclonal antibodies for 60min at 37°C, and then washed in PBS again. Biotin-conjugated secondary antibodies for 20min at 20–37°C, washed with PBS for four times, then stained by DAB Staining Kit, washed with water, and then counterstained with hematoxylin.

### Statistical analysis

The data were reported as mean±SD, and mean comparisons were performed by one-way ANOVA. P < 0.05 was accepted as statistically significant. Statistical analysis was performed using SPSS, v. 17.0.

## Results

### Cytotoxic effects of UA on BGC-823 cells

**Fig 1:**
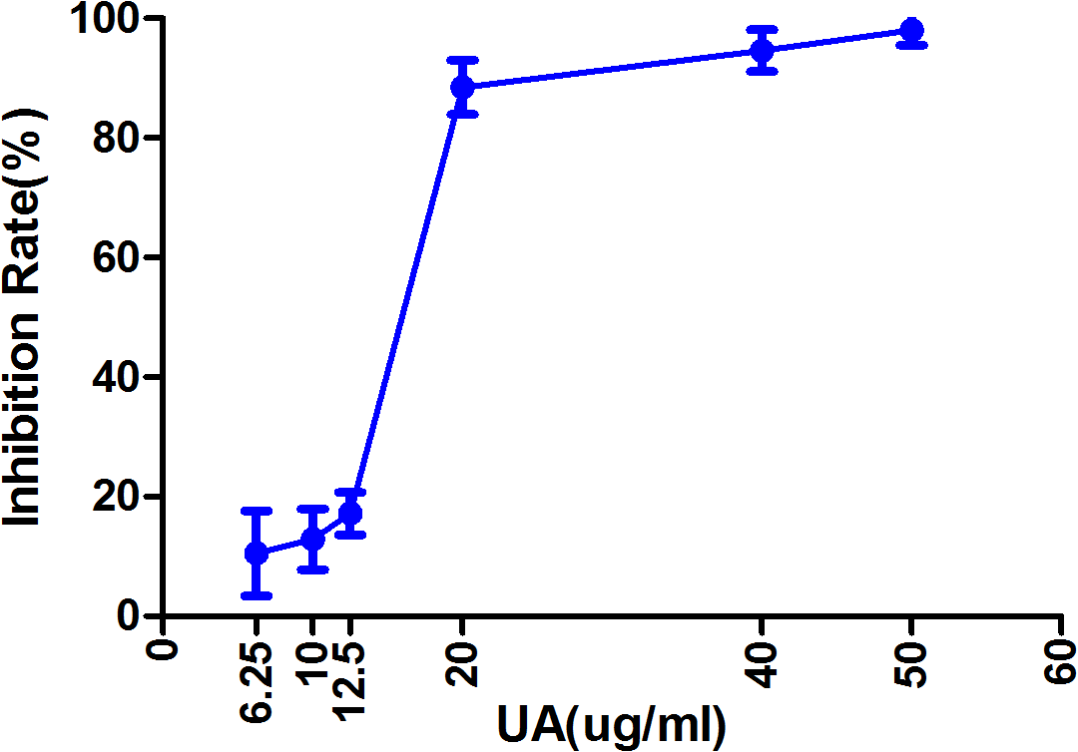
Cytotoxic effects of UA in BGC-823 cells. After 48h incubation with UA at the indicated concentrations, cytotoxicity was determined by the MTT assay. UA caused cytotoxicity in a dose-dependent manner, the IC_50_ of UA for BGC-823 was 17.5ug/ml, and we defined 10ug/ml as a slight cytotoxicity (sub-cytotoxicity) (<15%) concentration.

The cytotoxic effect of UA on BGC-823 cells was evaluated based on the MTT assay. We found that UA dose-dependently increased cytotoxicity in the gastric cancer cell line BGC-823 (Fig 1). The half-maximal inhibitory concentration (IC_50_) values for BGC-823 was 17.5ug/ml. Slight cytotoxicity (<15%) of UA for BGC-823, 10ug/ml, was used for subsequent planned experiments, to control for the radioenhancement due to a direct cytotoxic effect.

### The radiosensitization effects of UA in vitro

**Fig 2:**
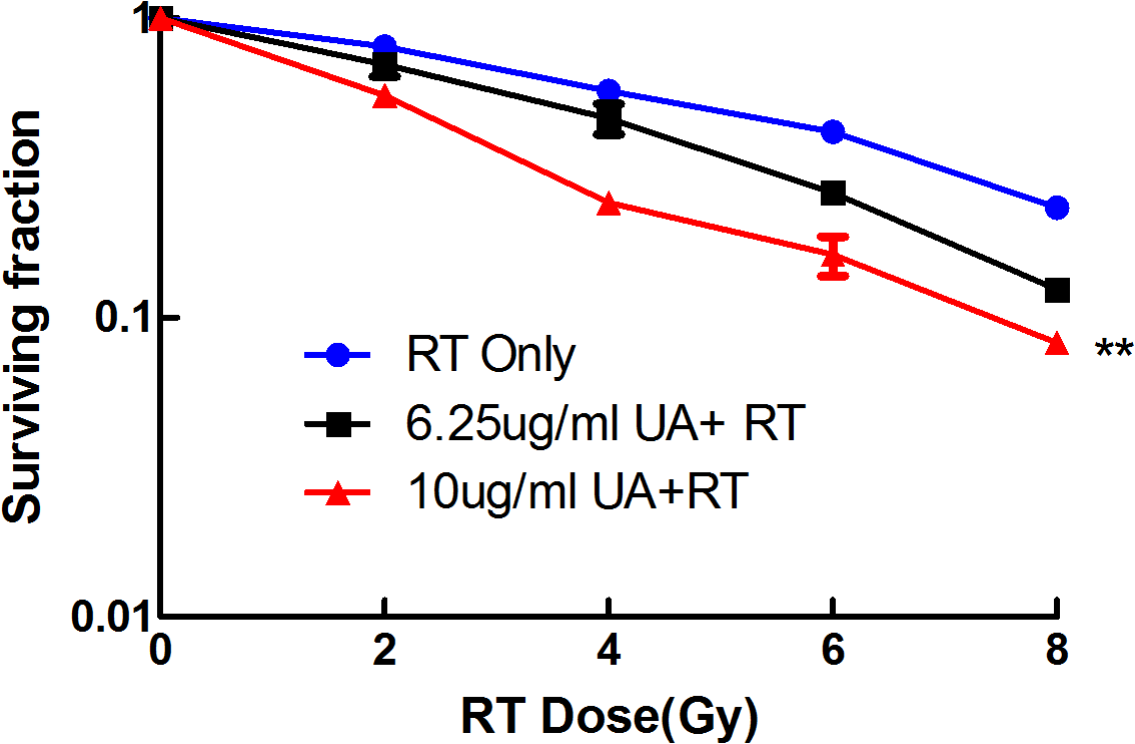
The radiosensitization effects of UA in BGC-823 cells. Radiosensitivity was determined by clonogenic survival assay. Surviving fraction of the combined group were significantly decreased compared with the irradiation group (** p<0.01).

Cell survival curves measured by clonogenic survival assay were illustrated in Fig 2. Compared with RT only, SF_2_ of RT combined with UA significantly decreased in BGC-823 cells, SER values of the combined groups were 1.180-fold and 1.315-fold. These results indicated that UA enhanced the radiation effect of BGC-823 in a dose-dependently manner within a slight cytotoxicity concentrations.

### Cell cycle distribution and induction of apoptosis.

**Fig 3:**
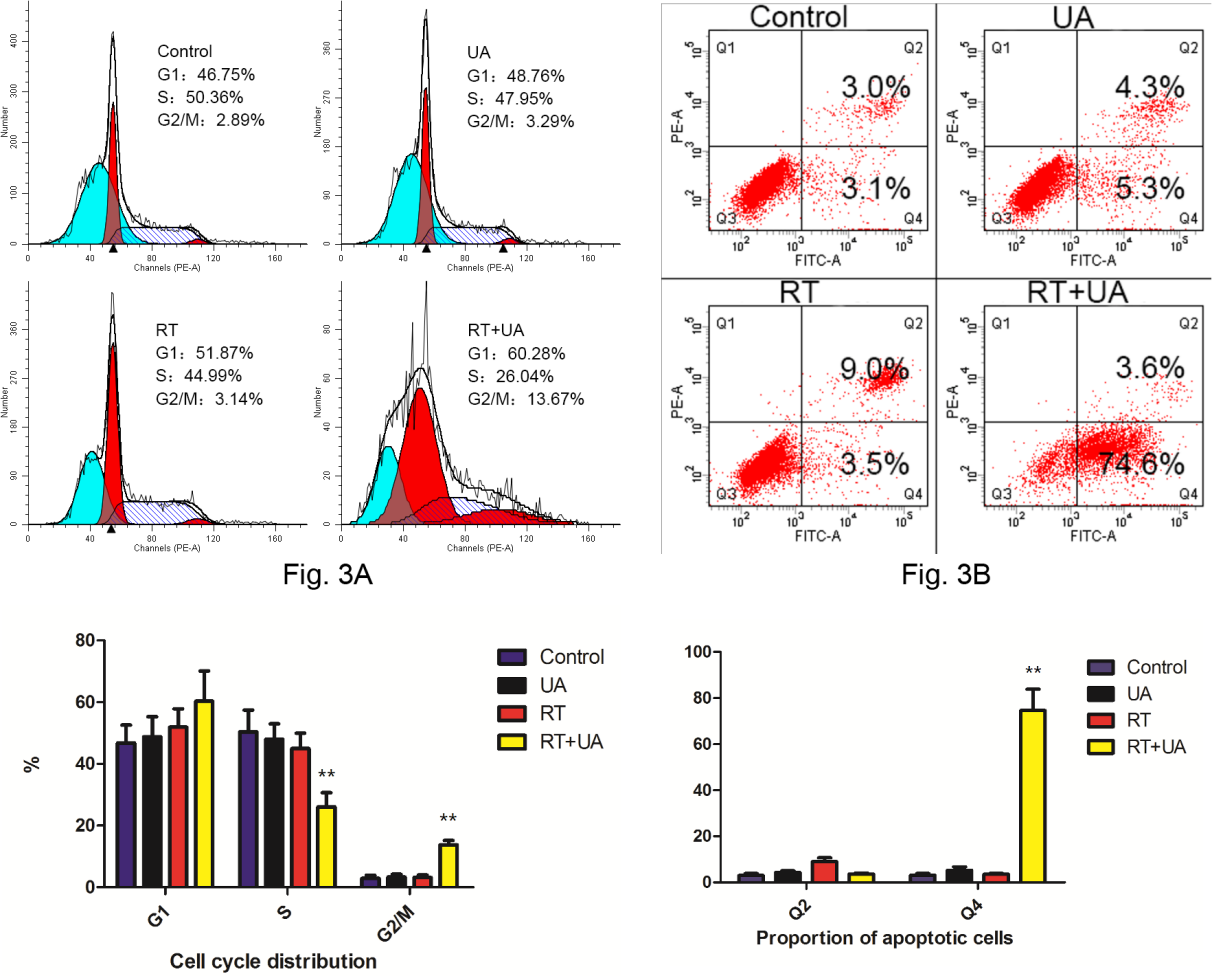
Effects of UA and radiation on cell-cycle progressing and apoptotic cell death of BGC-823 cells. DNA content of fixed and PI-stained cells was measured by flow cytometry, and cell-cycle was analyzed. Percentages of cells in each phase of the cell cycle were provided in each diagram (Fig 3A). Compared with IR group, the addition of UA induced cell cycle arrest at the G_1_ phase and G_2_/M phase and a significantly decline of S phase, demonstrating resistance to radiation therapy. Fig 3B showed the proportion of apoptotic cells (annexin V^+^, PI^+/-^). The combination group induced more apoptosis of BGC-823 cells compared with the other three groups.

To investigate whether RT and/or UA treatment could cause any perturbation of the cell cycle, an analysis of BGC-823 cells treated with 2Gy irradiation and/or 10ug/ml UA was performed. Relative to the control group, 10ug/ml UA alone yielded almost no effect on the cell cycle. However, the combined group of UA and irradiation induced cell cycle arrest at the G_1_ phase (51.87% VS 60.28%, p = 0.13) and the G_2_/M phase (3.14% VS 13.67%, p<0.01) compared with the 2Gy irradiation group (Fig 3A and 3C). These findings suggest that the combination treatment may have caused G_1_ phase and G_2_/M phase arrest, which was more susceptible to the damaging effects of radiation. The similar conclusions from the cell cycle data has been proven by previous study.[15]

The combination of UA and irradiation could also cause more apoptotic cell death as shown by Fig 3B. The proportion of apoptotic cells substantially increased by application of both UA and irradiation in BGC-823 cells as opposed to UA or IR treatment alone (78.2% VS 9.6% and 12.5%, p<0.01, Fig 3D).

### UA increased RT induced ROS formation

**Fig 4:**
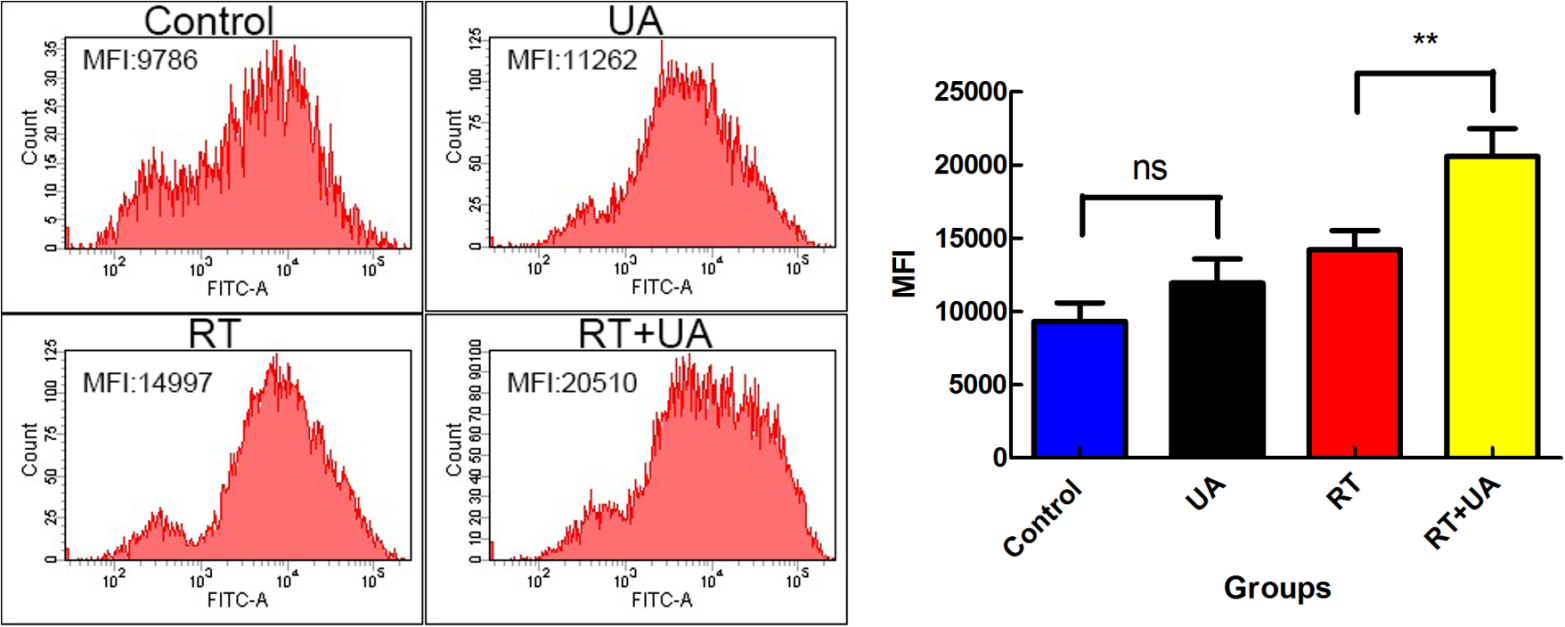
Effects of UA and radiation on ROS formation. The concentrations of intracellular ROS were detected using the membrane-permeable fluorescent probes 20, 70-dichlorofluorescin diacetate (DCFH-DA) by flow cytometry. The mean fluorescent intensity (MFI) represented intracellular ROS level. The MFIs difference between combination group and RT group was significant (P < 0.01) (ns: not significant; **:P < 0.01).

It is widely accepted that cell killing after exposure to ionizing radiation is partially mediated by ROS.[16] ROS analysis using DCF-DA produced the mean fluorescent intensity (MFI), which represented intracellular ROS level of each group (Fig 4A and 4B). MFIs of UA alone group, RT alone group and UA+RT group increased by 1.15-fold, 1.53-fold and 2.09-fold compared with the control group. The level of ROS of the combination group increased significantly relative to the RT alone group, revealing that adding UA to RT induced more ROS formation than RT alone.

### Immunohistochemical staining of Ki-67

**Fig 5:**
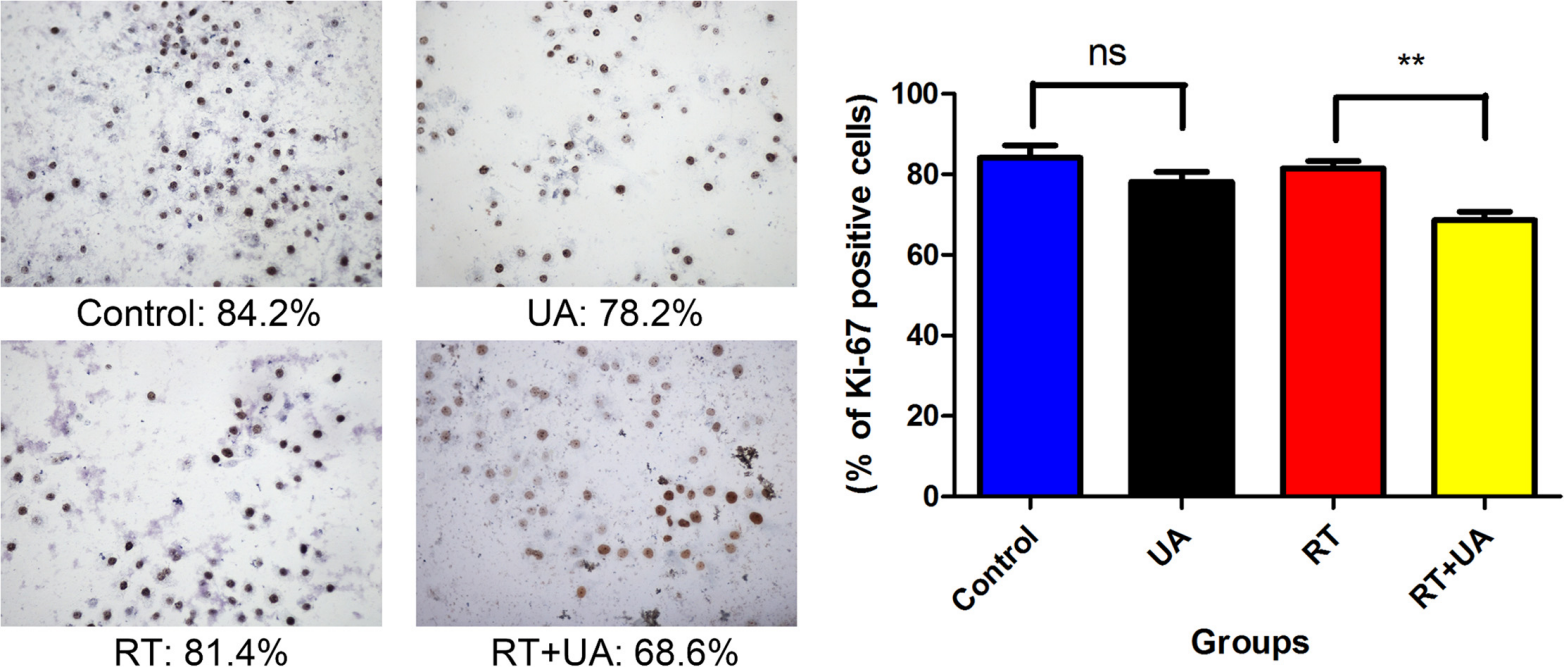
Effects of UA and radiation on the percentage of Ki-67 positive cells. Fig 5A showed the images of the results of Ki-67 immunohistochemical staining of BGC-823 cell line (magnification: 10 x 40). Percentage of Ki-67 positive cells of the combined group decreased significantly compared with the control group, as well as the radiation therapy group. Fig 5B showed that the difference between the control group and the UA group was non-significant; However, there was a significant decline between the combination group and the RT group (** P < 0.01).

Ki-67 was known as a nuclear antigen, which often correlates with cell proliferation. Our experiment detected the positive rate of Ki-67 protein by immunohistochemical staining, and the results were illustrated in Fig 5. Percentage of Ki-67 positive cells of the combined group decreased significantly compared with the control group, as well as the radiation therapy group.

## Discussion

This research examined the effects and possible mechanism of UA as a radiosensitizer. The MTT assay and clonogenic survival assay demonstrated that IR combined with UA was superior in cytotoxicity than IR alone. This effect was observed in both groups with different sub-cytotoxicity concentrations of UA, 6.25ug/ml and 10ug/ml. At this dose, UA had slight cytotoxicity to human adenocarcinoma gastric cancer cell line BGC823 and did not have significant cytotoxicity to normal gastric cell line.[17] This demonstrated that UA was a promising radiosensitization *in vitro*.

Central to cancer radiotherapy is maximizing therapeutic efficacy to tumor tissue while minimizing the damage to normal tissues. While elaborated planning and recent physical techniques may safely increase the radiation dose to tumor tissue while reducing the dose to surrounding normal tissues, [18] current radiotherapy techniques still cannot avoid injuring nearby normal tissues. Further radiotherapeutic benefit may be found by examining the biological response of the tumor microenvironment to uncover how to increase a tumor’s sensitivity to radiation or inhibit side effects by radiation.[19]

Normal tissues around gastric cancer, such as intestinal and gastric, are sensitive to ionizing radiation. As a result, the ionizing radiation dose towards tumor tissue must be restricted. Moreover, the overlapped side effects of ionizing radiation will enhance radiotherapy side effects, and even interrupt treatment. Sub-cytotoxicity concentration of UA combined with ionizing radiation, however, may increase the cytotoxicity by ionizing radiation, while low concentration UA is almost harmless to normal tissues. This strategy can increase the therapeutic index to tumor tissue without increasing radiation dose and radiotherapeutic side effect.

To identify the mechanism of radiosensitization of UA, we performed analysis in multiple steps. First, we analyzed the cell cycle distribution. Sub-cytotoxicity concentration of UA didn't yield any significant effect on the cell cycle, whereas compare with ionizing radiation alone, the combined treatment enhanced G_2_/M arrest. The radiosensitivity of cells is correlated to cell cycle, and the correlation is higher in the G_2_/M phase and lower in S phase. This may be the most important underlying reason for the enhanced radiosensitization observed in this study. Second, we analyzed the intracellular ROS levels. The ROS level of the combination group significantly increased compared with the control group, the UA alone group, or even the ionizing radiation group. The addition of UA to ionizing radiation induced more ROS formation. Ionizing radiation induced the radiolysis of water, which generated ROS, such as hydroxyl radicals and superoxide. These ROS molecules played an important role in ionizing radiation induced cellular lesions, such as oxidative DNA damage, potentially leading to both clonogenic death and apoptosis. Third, in this study, we found that percentage of Ki-67 positive cells of the combination group decreased significantly compared with the control group or the radiation therapy group. Ki-67 is known as a cell cycle associated antigen and a useful proliferation marker. Ki-67 protein level is correlated with higher proliferation and metastases ability.[20,21]

In conclusion, the present study is the first attempt to demonstrate that UA could synergize with ionizing radiation against human gastric cancer cell lines. The underlying mechanism of the improved radiosensitization efficacy may be the enhanced G2/M arrest, increased ROS and down-regulated Ki-67 level. Collectively, as UA demonstrated potent antiproliferation effect and synergistic effect, it could be used as a potential drug sensitizer for the application of radiotherapy. However, it is without a doubt that further research is needed to evaluate the feasibility and benefits of using Traditional Chinese Medicine as radiosensitizers before actual clinical applications. More experiments and replications are needed in other tumor types and in animal trials in order to confirm the efficacy of the proposed strategy.
